# Prevalence and location of inflammatory and structural lesions in patients with rheumatoid arthritis and radiographic axial spondyloarthritis with chronic neck pain evaluated by magnetic resonance imaging

**DOI:** 10.1186/s13075-024-03377-8

**Published:** 2024-07-25

**Authors:** David Kiefer, Mina Soltani, Parham Damirchi, Uta Kiltz, Bjoern Buehring, Ioana Andreica, Philipp Sewerin, Xenofon Baraliakos

**Affiliations:** 1 Ruhr-Universität Bochum, Bochum, Germany; 2 Rheumazentrum Ruhrgebiet, Herne Claudiusstr 45, 44649 Herne, Germany; 3 Bergisches Rheuma-Zentrum, Wuppertal, Germany; 4 Hiller Research Center, Medical Faculty, University Hospital Düsseldorf, Heinrich Heine University Düsseldorf, Düsseldorf, Germany; 5 Rheumazentrum Ruhrgebiet, Ruhr-University Bochum, Claudiusstrasse 45, 44649 Herne, Germany

**Keywords:** Axial spondyloarthritis, Rheumatoid arthritis, Osteoarthritis, Neck pain, MRI

## Abstract

**Objective:**

Define the prevalence and location of inflammatory and structural lesions on magnetic resonance imaging (MRI) in patients with rheumatoid arthritis (RA) and radiographic axial spondyloarthritis (r-axSpA) with neck pain as leading clinical symptom.

**Methods:**

Patients with diagnosis of RA and r-axSpA were consecutively included if they had chronic (> 3 months) neck pain. Clinical assessment, neck pain questionnaires and MRIs of the cervical spine (CS) were performed.

**Results:**

107 patients (59 RA and 48 r-axSpA) were included. While there was no difference in the Northwick-Park-Neck-Pain-questionnaire, patients with RA reported higher neck pain compared to r-axSpA on a numeric rating scale (5.0 ± 3.6 vs. 3.0 ± 3.1; *p* = 0.003). Inflammatory lesions occurred predominantly in the craniocervical area in RA and in the lower CS segments in r-axSpA. Bone marrow edema (BME) was more frequent in axSpA (BME-score axSpA/RA: 0.35vs0.17; *p* < 0.001) while synovitis was visible in both but was more prevalent in RA (synovitis-score axSpA/RA: 0.02vs0.1; *p* < 0.001). BME was found in 8 (13.6%) vertebral corner vs. 9 (18.8%), in 2 (3.4%) facet joints vs. 7 (14.6%) and in 1 (1.7%) spinous processes vs. 9 (18.8%) in patients with RA/r-axSpA. In contrast, more patients with RA (30.5% vs6.3%) showed erosive osteochondrosis with endplate BME (*p* = 0.002).

**Conclusion:**

While involvement of upper cervical inflammation was typically present in RA, r-axSpA patients showed more BME in lower CS segments, vertebral corners, facet joints and spinous processes. Neck pain is linked to upper and lower inflammatory and structural lesions of the CS in both diseases.

## Introduction

Rheumatoid arthritis (RA) and axial spondyloarthritis (axSpA) are both typical chronic inflammatory rheumatic diseases, sharing similarities like inflammation and structural lesions of joints; nevertheless, they diverge in terms of their pathophysiological pathways, clinical manifestations and the consequent structural and inflammatory findings in imaging [1].

Typical manifestations in RA are inflammations in the peripheral joints of hands and feet [1–3]. However, cervical spine (CS) involvement is the third most common manifestation of RA [4]. While the first and second cervical vertebrae (C1-C2) of the axial skeleton are often affected [5, 6], the thoracic and lumbar spine segments are spared in most RA patients [7–11]. While the atlantoaxial area is known to be affected in up to 20% of the patients with RA, other segments of the CS are less frequently affected [12]. On the other hand, typical imaging findings in axSpA are characterized by bone marrow edema (BME) in the axial skeleton [13]. Magnetic resonance imaging (MRI) is the gold standard assessment of inflammatory findings in both diseases. Depending on the presence or absence of definite structural lesions in the sacroiliac joints (SIJ) as assessed by conventional radiography – patients can be classified into a non-radiographic (nr-axSpA) and a radiographic form of axSpA (r-axSpA), the latter is largely equivalent to the classical ankylosing spondylitis (AS) [14–17].

Neck pain is a frequent clinical symptom in the general population and in patients with RA and r-axSpA and is associated with relevant functional impairment [3, 18–20]. Spinal symptoms may be a result of both inflammatory or structural lesions of the underlying disease or may be due to comorbidities such as degenerative disorders.

This prospective study aimed to compare the prevalence and pattern of inflammatory and structural findings obtained by MRI in patients with RA and r-axSpA who present with chronic neck pain, and to correlate these MRI findings with clinical measures and pain questionnaires.

## Materials and methods

For this prospective study, patients diagnosed with RA and r-axSpA were consecutively included if they reported neck pain with duration > 3 months. Inclusion was independent of the status of disease activity of the underlying disease.

### Clinical assessments and assessment of neck pain severity

Demographic data and disease characteristics were assessed in all patients. Laboratory markers C-reactive protein (CRP), erythrocyte sedimentation rate (ESR), rheumatoid factor (RF) and Human Leukocyte Antigen B27 (HLA B27) were taken from clinical routine. Disease specific assessments of disease activity were assessed using the patient global Assessment (PGA) of disease activity for all patients, the disease activity score 28 (DAS28) for patients with RA and the Bath ankylosing spondylitis disease activity index (BASDAI) for patients with r-axSpA [21]. Patients with r-axSpA were asked to complete the Bath ankylosing spondylitis functional index (BASFI). In addition, all participants underwent physical examinations including tragus-to-wall distance (in cm) and cervical rotation (angular degrees, °) were measured at both sides twice and mean values were calculated.

In addition, all patients filled in the Northwick Park Neck Pain questionnaire (NPQ). The NPQ measures the neck pain and provides an objective measure to evaluate outcome and monitor symptoms in patients with acute or chronic neck pain [22]. The NPQ comprises 9 items: (1) neck pain intensity, (2) neck pain and sleeping, (3) pins and needles or numbness in the arms at night, (4) duration of symptoms, (5) carrying, (6) reading and watching television, (7) working and/or housework, (8) social activities and (9) driving. Each of the 9 items features 5 ordinal responses on a Likert scale ranging from 0 to 4; higher scores indicate greater levels of pain or disability. Responses to these questions are summed and divided by 36 to give a percentage score. Question 9 relates to driving, and if this is not applicable the total score is instead divided by 32. The NPQ has a good short-term reliability, a high internal consistency and responsiveness [23, 24]. Furthermore, all patients completed following 2 questions (Q) to characterize the severity (numeric rating scales 0–10) and duration of the neck pain (years): Q1. “Please rate the severity of your neck pain over the past 7 days on a scale from 0 to 10, with 10 indicating the worst possible pain.” And Q2. “Since how many years have you been experiencing this neck pain?“. Responses for Q2 were given in specific number of years indicating the duration of neck pain.

### MRI examinations and evaluation

All patients prospectively underwent cervical MRI examinations on a 1.5 Tesla machine (Siemens Magnetom) using a phased array neck coil. The following sequences were acquired: sagittal and coronar T1- weighted ((T1w) turbo spin-echo, thickness 3 mm), sagittal and transversal T2-weighted ((T2w) turbo spin-echo, thickness 3 mm). After injection of intravenous contrast agent, the following sequences were acquired: sagittal, coronar and transversal fat-suppressed T1w (turbo spin-echo thickness 3 mm). Images were viewed on certified Dicom-compliant workstations.

Two readers (MS; PD), both specialist radiologists with experience in reading musculoskeletal MRI scans, blinded for demographics and diagnosis, evaluated MRIs including binary notification (presence or absence) of inflammatory or structural lesions. The latter included inflammation (BME or synovitis) and structural lesions of the upper and lower CS. Inflammation was defined as BME or synovitis. The MRI scoring system of Supphia et al. quantifies BME, synovitis, and erosions in the CS with good reliability and feasibility and has been reported previously [25, 26]. Pathologies were considered positive if identified by both readers (MS; PD) or, in case of disagreement of the MRI findings, if confirmed by a third, experienced reader who served as for adjudication (XB).

Inflammation was assessed based on the presence of Bone Marrow Edema (BME) or synovitis. Additionally, the presence of synovitis (atlantooccipital, atlantoaxial, and atlantodental) and BME in the vertebral corners, facet joints, and spinous process were also scored. For quantification of BME and synovitis a modified version of the previously published MRI scoring system of Supphia R. et al. was used as a basis [25, 26]. The observations were quantified by concentrating on the extension of BME and synovitis in the CS. The vertebral bodies of C1–C7 were scored as follows: 0 (BME < 10% of the bone surface), 1 (10–33% of the bone surface), 2 (34–66% of the bone surface), and 3 (> 66% of the bone surface). For C1, the anterior arch substituted for the body. The same grading of 0–3 was used for the spinous process and for the facet joints, with the right and the left facet joint of C2–C7 being scored together. The dens of C2 was considered an additional component. Each vertebral component was semiquantitatively scored 0–3 based on the estimated volume of BME present (where score 0 = no BME; 1 = < 33% BME; 2 = 33–66% BME; and 3 = > 66% BME. The maximum possible score for BME was 69.

The presence of synovitis was assessed in three specific areas: (1) The atlantooccipital joints, (2) the lateral atlantoaxial joints (combining right and left) and (3) the medial atlantoaxial joint (or atlantodental joint). A score of 0 denoted the absence of synovitis, while a score of 1 indicated the presence of synovitis, with a maximum possible score of 3.

The scoring of structural lesions was based on the presence of specific pathologies, including erosions as postinflammatory lesions in the dens-axis region, as well as those predominantly associated with degenerative disorders such as erosive osteochondrosis [27]. Erosive osteochondrosis was defined similar to previous studies [28, 29] as the irregular cortical bone outline of the endplates observed on MR images, accompanied by a low signal of the adjacent intervertebral disc on T2-weighted sequences.

Protrusions were detected when the intervertebral disc extended beyond its normal boundaries. Disc herniation was assessed when the inner material of the intervertebral disc leaked out through a tear in the disc’s outer layer. Spondylosis was defined degenerative lesions involving wear and tear of the spinal discs and osteoarthritis of the facet joints was defined structural lesions in the joints connecting adjacent vertebrae in the spine [28].

This study was approved by the Ethical Committee of the Medical Council of Münster Westphalia, reference number 2013-107-f-S. Written informed consent was obtained from all patients.

### Statistical analysis

Mean values, standard deviations and frequencies are provided descriptively. Comparisons between groups were performed using the t-Test for independent samples for the mean values and using the chi-square test for frequencies. The correlations between clinical and imaging outcomes were calculated with Spearman´s correlation coefficient.

A p-value < 0.05 was considered statistically significant.

Statistical analysis was performed with SPSS software, v. 26.0.

## Results

### Demographic and clinical characteristics

A total of 107 patients were included in the study, comprising 59 with RA (55.1%), and 48 (44.9%) with r-axSpA and 413 vertebral segments (RA) and 336 vertebral segments (r-axSpA) were finally evaluated. RA patients were more likely women (*n* = 39; 66,1%, *p* < 0.001), while more men were included in the r-axSpA group (*n* = 33; 68,8%). In RA, the mean age was higher compared to r-axSpA (58.6 ± 11.4 years vs. 47.9 ± 13.1 years, *p* < 0.001) while RA patients had a shorter disease duration (6.7 ± 6.8 vs. 10.2 ± 12.8; *p* = 0.430). Patients with RA presented a moderate disease activity (mean DAS28: 3.9 ± 1.9), patients with r-axSpA presented an active disease (BASDAI: 4.6 ± 1.8), the patient global assessment (PGA) was comparable in both groups (Table 1) Functional impairments in cervical spinal mobility, measured by Tragus to wall distance and cervical rotation were comparable in both groups (13.9 ± 3.3 cm; 48.4 ± 20.0°) and r-axSpA (14.3 ± 4.8 cm, *p* = 0.84; 45.4 ± 21.4°, *p* = 0.84) and. Serological markers for inflammation CRP and ESR, were also comparable.

**Table 1:** Patients and disease characteristics

	RA (*n* = 59)	R-axSpA (*n* = 48)	*p*-value
Age (years)	58.6 (11.4)	47.9 (13.1)	< 0.001
Female, n (%)	39 (66.1)	15 (31.2)	< 0.001
Disease duration (years)	6.7 (6.8)	10.2 (12.8)	0.430
Q2: Duration of neck pain (years)	6.4 (9.8)	6.9 (11.0)	0.274
Rheumatoid factor n (%)	28 (47.5)	n.a.	-
HLA B27 positive; n (%)	n.a.	32 (69.6)	-
Neckpain, NRS (0–10)	5.0 (3.6)	3.0 (3.1)	0.003
Northwick neck pain questionnaire, %	32.0 (20.5)	35.9/24.4	0.143
CRP (mg/dl)	1.2 (2.3)	0.9 (1.4)	0.907
ESR (mm/h)	19.4 (16.9)	15.7 (18.1)	0,084
PGA	6.3 (1.9)	6.2 (2.2)	0,881
DAS28	3.9 (1.4)	n.a.	-
BASDAI	n.a.	4.6 (1.8)	-
BASFI	n.a.	4.7 (2.6)	-
Tragus-to-wall distance, cm	13.9 (3.3)	14.3 (4.8)	0.84
Cervical Rotation, degree	48.4 (20.0)	45.4 (21.4)	0.45

*Variables are mean ± standard deviation if not otherwise indicated. R-axSpA: radiographic-axial spondyloarthritis; RA: rheumatoid Arthritis; BASMI: Bath Ankylosing Spondylitis (AS) Metrology Index; BASDAI: Bath AS Disease Activity Index; BASFI: Bath AS Functional Index; CRP: C-reactive protein; ESR: erythrocyte sedimentation rate; NSAIDs: Non-steroidal anti-inflammatory drugs; NRS = numerical rating scale; HLA B27: Human Leukocyte Antigen-B 27; DAS28: Disease activity score 28; PGA: Patient global assessment. Q = Question.

When comparing neck pain, RA patients reported significant higher mean levels of neck pain (Q1) as compared to r-axSpA patients (5.0 ± 3.6 vs. 3.0 ± 3.1; *p* = 0.003), while the duration of neck pain was comparable (Table 1). On the other hand, the NPQ revealed no differences as demonstrated in Table 1 (RA: 32.0 ± 20.5 vs. r-axSpA: 35.9 ± 24.4; *p* = 0.143).

### Imaging outcomes

**Fig. 1:**
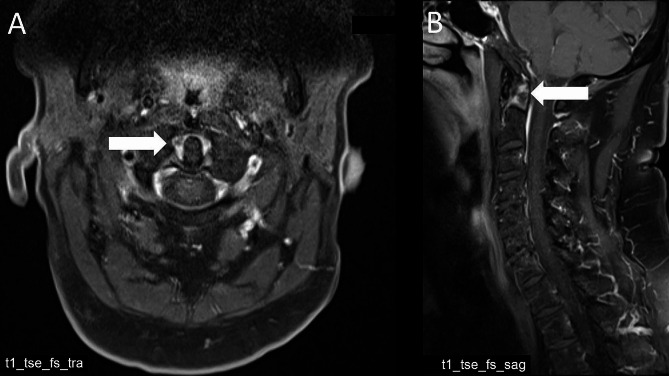
Atlantodental synovitis. a Transversal and b sagittal MRI (T1- weighted with contrast agent), showing atlantodental synovitis (arrow) of a patient with rheumatoid Arthritis

The pattern of CS inflammation differed between patients with RA and r-axSpA (Table 2). Compared to patients with r-axSpA, inflammatory changes (BME and synovitis) in RA patients were mostly present in the cranio-cervical segments (Table 2). In detail, atlantoaxial synovitis was found in 5 (8.5%) patients with RA but in only 1 (2.1%) patient with r-axSpA (*p* = 0.255) and atlantodental synovitis (Fig. 1) in 5 (8.5%) RA patients but in none of the r-axSpA patients (*p* = 0.040).

**Table 2:** Inflammatory findings on magnetic resonance imaging (MRI) in patients with RA and r-axSpA.

Inflammatory MRI changes*	RA (*n* = 59)	R-axSpA (*n* = 48)	*p*-value
Atlantookzipital synovitis	0	0	1
Atlantoaxial synovitis, n (%)	5 (8.5)	1 (2.1)	0.255
Atlantodental synovitis, n (%)	5 (8.5)	0	0.040
BME present in any location, n (%)	9 (15.3)	11 (22.9)	0.166
BME at area of vertebral corner, n (%)	8 (13.6)	9 (18.8)	0.467
BME in the facet joints, n (%)	2 (3.4))	7 (14.6)	0.039
BME in the spinous process, n (%)	1 (1.7)	9 (18.8)	0.003
BME score (0–66)	0.17 (0.56)	0.35 (0.98)	< 0.001
Synovitis score (0–3)	0.1 (0.36)	0.02 (0.14)	< 0.001

*Variables are mean ± standard deviation if not otherwise indicated; MRI: Magnet resonance imaging; R-axSpA: radiographic-axial spondyloarthritis; RA: rheumatoid Arthritis; BME: Bone marrow edema

**Fig. 2:**
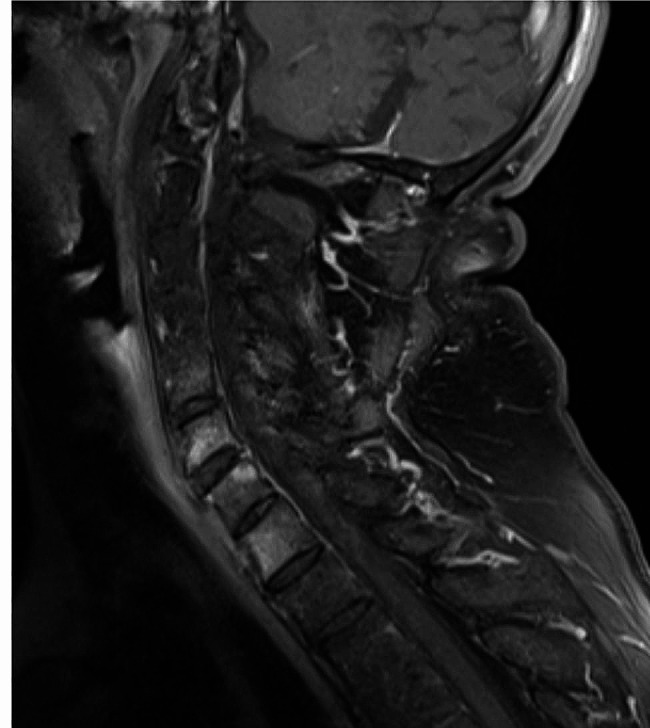
Bone marrow edema in the vertebral bodies. Sagittal MRI (T1- weighted with contrast agent), showing bone marrow edema in the vertebral bodies, mainly located in the area of the vertebral corner (arrow) of a patient with ankylosing spondylitis

On the other hand, BME in CS of patients with r-axSpA were mainly found in lower segments (Fig. 2). There were numerically more patients with r-axSpA (*n* = 11, 22.9%) than RA (*n* = 9, 15.3%; *p* = 0.166) with BME. In patients with r-axSpA, BME was localized in 9 patients (18.8%) at the area of vertebral corner, in 7 patients (14.6%) in the facet joints and in 9 patients (18.8%) in the spinous processes, compared to 8 patients (13.6%) at the vertebral corner and only in 2 patients (3.4%) in the facet joints and in 1 patient (1.7%) in the spinous process with RA (Table 2). There was significant more BME detected in the facet joints (*p* = 0.039) and in the spinous processes (0 = 0.003) in patients with r-axSpA than in RA (Table 2). When using the BME and synovitis scoring system of Suppiah we see significant differences between the diseases. While there is more BME in r-axSpA, more synovitis could be detected for RA (both *p* < 0.0001). In contrast to MRI findings of inflammation, erosive lesions in the dens-axis region were found in both groups, but surprisingly numerically more in patients with r-axSpA (5; 10.4%) than RA (3; 5.1%) patients but without a statistically significant difference (*p* = 0.824; Table 3).

**Table 3:** Structural findings on magnetic resonance imaging (MRI) in patients with RA and r-axSpA

Structural MRI lesions*	RA (*n* = 59)	AS (*n* = 48)	*p*-value
Erosive osteochondrosis, n (%)	18 (30.5)	3 (6.3)	0.011
Protrusion, n (%)	54 (91.5)	30 (62.5)	< 0.001
Disc herniation, n (%)	24 (40.7)	9 (18.8)	0.015
Spondylosis, n (%)	40 (67.8)	21 (43.8)	0.013
Facet joint osteoarthritis, n (%)	46 (78.0)	31(64.6)	0.127
Erosive lesions in the dens-axis region, n (%)	3 (5.1)	5(10.4)	0.824

*Variables are mean ± standard deviation if not otherwise indicated; MRI: Magnet resonance imaging; R-axSpA: radiographic-axial spondyloarthritis; RA: rheumatoid Arthritis

Finally, in the analysis of structural lesions in patients with RA showed to have significant more lesions in most locations (Table 3). In detail, significant more patients with RA presented erosive osteochchondrosis (30.5% vs. 6.3%), protrusions (91.5% vs. 62.5%), disc herniations (40.7,8%vs.18.8%), and spondylosis (67.8% vs. 43.8%) spondylosis (all *p* < 0,015). Numerically more patients with RA presented facet joint osteoarhtritis (*n* = 46, 78%) as compared to patients with r-axSpA (*n* = 31, 64.6%) but this result was not statistically significant (*p* = 0.127) (Table 3).

### Association between clinical and imaging outcomes

In RA there was no correlation between disease activity (DAS28) and any inflammatory or structural pathology on MRI. CRP had weak correlations with atlantodental synovitis (*r* = 0.292; *p* = 0.025) and atlantoaxial synovitis (*r* = 0.264; *p* = 0.043) while ESR correlated with erosive lesions in the dens-axis region (*r* = 0.485; p = < 0.001). As expected, positive correlations could be found for age and structural lesions in the MRI but not for disease duration and duration of neck pain (Table 4). For measurements of spinal cervical mobility almost no correlations could be fund except the number of protrusions correlated with impairment of cervical rotation and the presence of protrusion as well as the number of facet joint osteoarthritis correlated with impairments in tragus to wall distance (Table 4). For neck pain and the NPQ weak correlations were found with atlantodental synovitis (*r* = 0.295; p = < 0.024 and *r* = 0.321; p = < 0.013) and for neck pain also with facet joint osteoarthritis (*r* = 0.259; p = < 0.049).

**Table 4:** Correlations of demographic and clinical outcomes with imaging outcomes in patients with rheumatoid arthritis

	Sex, female	Age (years)	Disease duration (years)	Duration of neck pain (years)	Neck pain. (NRS: 0–10)	NPQ	Tragus-to-wall distance. (cm)	Cervical Rotation. (degree)
Dens_BME (yes/no); r	0.26*	n.s.	n.s.	n.s.	n.s.	n.s.	n.s.	n.s.
Erosive lesions in the dens-axis region (yes/no); r	n.s.	0.29*	n.s.	n.s.	n.s.	n.s.	n.s.	n.s.
Atlantodental synovitis, r	n.s.	-0.31*	n.s.	n.s.	-0.30*	-0.32*	n.s.	0.33*
Atlantoaxial synovitis (yes/no); r	n.s.	n.s.	n.s.	n.s.	n.s.	n.s.	n.s.	n.s.
BME present in any location cervical spine (yes/no); r	n.s.	n.s.	n.s.	n.s.	n.s.	n.s.	n.s.	n.s.
Osteochondrosis Present (yes/no); r	n.s.	0.41**	n.s.	n.s.	n.s.	n.s.	n.s.	n.s.
Erosive osteochondrosis present (yes/no); r	n.s.	n.s.	-0.33*	n.s.	n.s.	n.s.	n.s.	n.s.
Facet joint osteoarthritis (yes/no); r	0.29*	0.37*	n.s.	n.s.	0.26*	n.s.	n.s.	n.s.
Number of Protrusion; r	n.s.	0.39*	n.s.	n.s.	n.s.	n.s.	0.30*	n.s.
Number of disc herniations; r	n.s.	n.s.	n.s.	n.s.	n.s.	n.s.	n.s.	n.s.
Protrusions present (yes/no); r	n.s.	0.47**	n.s.	n.s.	n.s.	n.s.	n.s.	-0.38*
disc herniations present (yes/no); r	n.s.	n.s.	n.s.	-0.27*	n.s.	n.s.	n.s.	n.s.
Number of osteochondrosis; r	n.s.	0.42**	n.s.	n.s.	n.s.	n.s.	n.s.	n.s.
Number of Spondylosis; r	n.s.	0.28*	n.s.	n.s.	n.s.	n.s.	n.s.	n.s.
Number of facet joint osteoarthritis ; r	0.33*	0.363*	n.s.	n.s.	n.s.	n.s.	n.s.	-0.29*
BME in the facet joints (yes/no); r	n.s.	n.s.	n.s.	n.s.	n.s.	n.s.	n.s.	n.s.

R = correlation coefficient; *: p-values ≤ 0.05; **: p-values ≤ 0.001; ns: not statistically significant; BME: Bone marrow edema; NRS: Numeric Rating Scale; NPQ: Northwick Park Neck Pain Questionnaire. N.s.: not significant

In r-axSpA there was no correlation between disease activity (BASDAI) and any inflammatory or structural pathology. ESR showed no correlation with imaging pathologies of inflammation, while CRP showed only a weak correlation with atlantoaxial synovitis (*r* = 0.294; *p* = 0.043). Similar to RA, no clinically relevant correlations with imaging outcomes and medication could be seen. As expected, positive correlations could be found for age and structural lesions in the MRI but not for inflammatory changes. Disease duration only correlated with the number of facet joint osteoarthritis (*r* = 0.434; *p* = 0.002) while duration of neck pain correlated with erosive lesions in the dens -axis region (*r* = 0.617; p = < 0.001), number of spondylosis (*r* = 0.305; *p* = 0.002) and facet joint osteoarthritis (*r* = 0.553; p = < 0.001) (Table 5).

**Table 5:** Correlations of demographic and clinical outcomes with imaging outcomes in patients with r-axSpA

	Sex (female)	Age (years)	Disease duration (years)	Duration of neck pain (years)	Neck pain (NRS)	NPQ	Tragus-to-wall distance (cm)	Cervical rotation (degree)
Dens_BME (yes/no), r	n.s.	n.s.	n.s.	n.s.	n.s.	n.s.	n.s.	n.s.
erosive lesions dens-axis (yes/no), r	n.s.	n.s.	ns	0.62**	n.s.	n.s.	0.43*	n.s.
Atlantodental Synovitis (yes/no), r	n.s.	n.s.	n.s.	n.s.	n.s.	n.s.	n.s.	n.s.
Atlantoaxial Synovitis (yes/no), r	n.s.	n.s.	n.s.	n.s.	n.s.	n.s.	0.34*	n.s.
BME present in any location cervical spine (yes/no), r	n.s.	n.s.	n.s.	n.s.	n.s.	n.s.	n.s.	n.s.
Osteochondrosis Present (yes/no), r	n.s.	n.s.	n.s.	n.s.	n.s.	n.s.	n.s.	n.s.
Erosive osteochondrosis present (yes/no), r	n.s.	0.5**	n.s.	n.s.	0.42*	0.47**	n.s.	-0.47**
Facet joint Osteoarthritis (yes/no), r	n.s.	0.29*	n.s.	n.s.	n.s.	n.s.	n.s.	n.s.
Number of protrusion, r	n.s.	0.36*	n.s.	n.s.	n.s.	0.37*	n.s.	n.s.
Number of disc Herniations, r	n.s.	n.s.	n.s.	n.s.	n.s.		n.s.	n.s.
Protrusions Present, (yes/no), r	n.s.	0.38*	n.s.	n.s.	n.s.	0.36*	n.s.	n.s.
Disc herniations Present (yes/no), r	n.s.	0.36*	n.s.	n.s.	n.s.	n.s.	n.s.	n.s.
Number of Osteochondrosis, r	n.s.	0.41*	n.s.	n.s.	n.s.	0.29*	n.s.	n.s.
Number of spondylosis, r	n.s.	0.64**	n.s.	0.31*	0.36*	0.43*	n.s.	-0.47**
Number of facet joint osteoarthritis, r	n.s.	n.s.	0.43*	0.55**	n.s.	n.s.	n.s.	n.s.

R = correlation coefficient; *: p-values ≤ 0.05; **: p-values ≤ 0.001; ns: not statistically significant; R-axSpA: radiographic-axial spondyloarthritis; BME: Bone marrow edema; NRS: Numeric Rating Scale; NPQ: Northwick Park Neck Pain Questionnaire. N.s.: not significant

For measurements of spinal cervical mobility almost no correlations could be found except the number of protrusions correlated with impairment of cervical rotation and the presence of protrusion as well as the number of facet joint osteoarthritis correlated with impairments in tragus to wall distance (Table 4).

In contrast to RA, neck pain and the NPQ showed no correlations with inflammatory changes but with structural lesions in patients with r-axSpA. In detail, neck pain correlated with the presence of erosive osteochondrosis (*r* = 0. 416; p = < 0.003) and the number of spondylosis (*r* = 0.358; p = < 0.012) and the NPQ also with presence and number of erosive osteochondrosis (*r* = 0.47; p = < 0.001; *r* = 0.293; p = < 0.043), presence and number of protrusions (*r* = 0.36; p = < 0.0012; *r* = 0.367; p = < 0.01) and number of spondylosis (*r* = 0.434; p = < 0.002) (Table 5).

## Discussion

To our knowledge, this is the first study that prospectively examined and compared the frequency and the pattern of both inflammatory and structural lesions of the CS in patients diagnosed with RA or r-axSpA who present with the leading clinical symptom of neck pain. While both RA and r-axSpA are recognized for their potential to manifest inflammatory and structural alterations in the CS, our findings reveal distinct patterns of involvement that distinguish these two conditions. Notably, this investigation provides prospective insights into the CS pathologies encountered in the context of chronic neck pain among patients with RA or r-axSpA.

Our study demonstrated a greater occurrence of synovitis in patients with RA while patients with r-axSpA exhibited a higher prevalence of BME. Inflammation in the more cranial regions of the CS such as atlantodental and atlantoaxial synovitis was typically found in patients with RA, while patients with r-axSpA presented with more inflammatory changes in the vertebral bodies, facet joints and the spinous process. Furthermore, erosive lesions in the dens-axis region, a finding considered typical in RA but not yet frequently described in r-axSpA, were found in both diseases but were overall rare as compared to other pathologic findings. Overall, our findings show that both diseases may lead to inflammation in both the upper and lower CS and that, contrary to common belief, localization of lesions is not strictly associated and pathognomonic to the respective disease. In addition, the presence of erosive changes in the dens axis region in r-axSpA may be of special clinical interest as these pathologies were widely considered a hallmark of RA [4].

In addition to the description of lesions, our results confirm recent findings where BME lesions detected by MRI [4] were not linked to disease duration, while, in contrast to our results, other studies demonstrated that structural antlantodental lesions in RA, are associated with a longer disease duration [30].

Neck pain may lead to mobility restrictions of the CS irrespective of the diagnosis, something that underlines the necessity for describing not only inflammatory but also structural lesions in imaging examinations as detailed as possible. These findings are also interesting from the explanation of cases of patients with r-axSpA with mobility restriction but no visible anterior syndesmophytes. As neck pain and the NPQ are mainly related to structural lesions, imaging plays a crucial role in the explanations of these findings.

Except for the finding of atlantodental synovitis in RA patients, our findings are consistent with previous research, indicating that neck pain does not necessarily have to correlated with MRI-detected inflammation [26]. In our study, despite the fact that all patients were included due to a leading clinical symptom of (chronic) neck pain, not all of them had an MRI lesion explaining this symptom.

Regarding the assessed PROs of neck pain, it is of interest that there is a discrepancy between the reported NRS scores for neck pain and the outcomes derived from the NPQ. An explanation for this finding might be that while the NPQ is primarily designed for assessing unspecific or degenerative neck pain, its ability to reflect the inflammatory components of patients with RA and r-axSpA may be limited, especially in association with the presence of inflammatory MRI lesion.

The early detection of the CS involvement is of high importance as, besides pain, it may lead to craniocervical and atlantoaxial instability, which can cause neural deficits and even death [31]. As new data show that, due to improvement of pharmaceutical treatment and treat to target strategies in recent years, there is decreasing prevalence [31, 32], even the treatment with biologics cannot fully prevent cervical involvement in RA [33]. Furthermore, evidence suggests that cervical involvement in axSpA is slightly more predominant in women compared to men; therefore, investigating the cervical spine may be beneficial for diagnosis, particularly given that women often experience longer diagnostic delays [34–37].

As a limitation of the study, it is important to highlight the absence of radiographic imaging modalities, such as x-ray or computed tomography (CT), which could have supported the findings of chronic lesions, particularly atlanto-axial dislocations or erosions in RA and syndesmophytes in r-axSpA. CT of the spine in axSpA is currently seen as an alternative imaging technique that allows comprehensive assessment of structural damage across all spinal segments [38–41]. CT-based measurements have shown to be superior to conventional radiography and MRI in detecting structural damage MRI [42, 43]. Therefore, CT is valuable in clinical trials and for distinguishing differential diagnoses. However, MRI remains the preferred imaging technique in routine clinical practice due to its ability to visualize both structural changes and inflammation [44, 45]. Moreover, CT, even at low or ultra-low doses, still involves radiation exposure [45]. Furthermore, this study did not examine the occurrence of lesions in patients without neck pain, something which is of similar clinical importance as the results presented here. Nevertheless, such data are available in the literature otherwise [46, 47]. The early detection of the CS involvement is of high importance as, besides pain, it may lead to craniocervical and atlantoaxial instability, which can cause neural deficits and even death [31]. This is of interest also since new data show that, despite the improvement of pharmaceutical treatment and treat-to-target strategies in recent years with decreasing prevalence of CS involvement under such treatment [31, 32], cervical involvement is a clinical issue that is still considered as being difficult to treat [33]. Finally, one could acknowledge as a limitation that we did not consider the different treatments as differentiating factor to the lesions observed. However, this information was not collected on purpose since our aim was to examine the imaging lesions in relation to neck pain which was present and was obviously independent of treatment. We therefore do not believe that this should be considered as something that is limiting largely the interpretation of our analysis.

## Conclusion

In summary, cervical involvement is not limited to one specific rheumatologic condition while the presence of atlantodental involvement is not an exclusive hallmark of RA but can also be found in patients with r-axSpA and chronic neck pain.

Future studies might explore the complementary role of various imaging modalities, including x-ray and CT, in diagnosing and monitoring CS involvement. Additionally, investigations could delve into the long-term impact of these findings on treatment strategies and patient outcomes.

## Data Availability

No datasets were generated or analysed during the current study.
